# A curious case of plant material emerging from a Mohs micrographic surgical site

**DOI:** 10.1016/j.jdcr.2025.07.028

**Published:** 2025-08-27

**Authors:** Kylee J.B. Kus, John L. McAfee, Steven D. Billings, Jennifer Lucas

**Affiliations:** a Department of Dermatology, Cleveland Clinic, Cleveland, Ohio; b Department of Pathology, Cleveland Clinic, Cleveland, Ohio

**Keywords:** botanical debris, case report, environmental, foreign material, Mohs micrographic surgery, plant matter, wound site

## Introduction

Foreign bodies may be retained in the body due to various causes such as ingestion, trauma, or surgical errors. Although skin puncture wounds or injuries are quite common, cases of retained foreign bodies are relatively rare.^1^ Here, we present a case of an unexpected foreign body following Mohs micrographic surgery to treat basal cell carcinoma.

## Case report

A 69-year-old woman presented to the dermatology clinic for a lesion of concern on her forehead that she had noticed 8 weeks prior. She stated that she had hit her forehead on the corner of a cabinet and that the subsequent lesion was not healing and would occasionally bleed. Physical examination revealed a 0.6-cm pink papule with rolled borders and an ulcerated center. A shave biopsy was taken, which resulted in basal cell carcinoma, including superficial-multifocal and nodular types.

Nearly 3 months later, the patient presented for Mohs micrographic surgery to treat the basal cell carcinoma. The lesion was cleared with a single layer and closed in a primary linear fashion with a deep layer of absorbable 5-0 Monocryl sutures and a top layer of nonabsorbable 6-0 Prolene sutures. Mohs micrographic surgery confirmed that the surgeon had gotten beneath the biopsy site, and there were no notable findings reported. One week later, the patient presented for suture removal. The wound was healing well with minimal erythema along the incision line. No signs of infection were observed, and the top sutures were easily removed.

**Fig 1:**
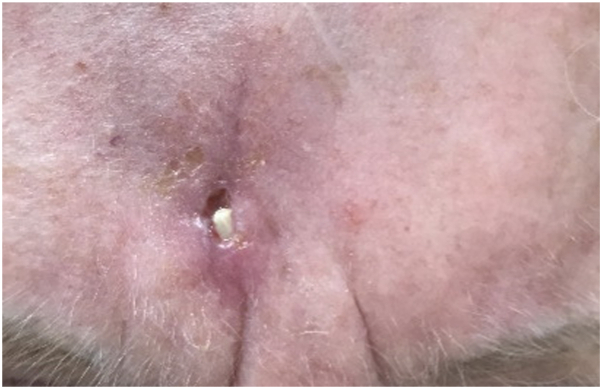
Clinical appearance of hardened, fibrous tissue, emerging from the incision site.

After a month, the patient returned to the clinic for a wound check. The surgical site exhibited slight dehiscence with hardened, fibrous tissue emerging from the incision site (Fig 1). She reported a mild pressure sensation in the area. A wound culture was collected, and an attempt was made to gently remove the fibrous tissue; however, it remained firmly in place. The wound culture grew moderate *Staphylococcus aureus*, and the patient was started on doxycycline 100 mg twice daily for 1 week. At subsequent follow-up, a shave biopsy was taken of the fibrous tissue to rule out a foreign body or keratin.

**Fig 2:**
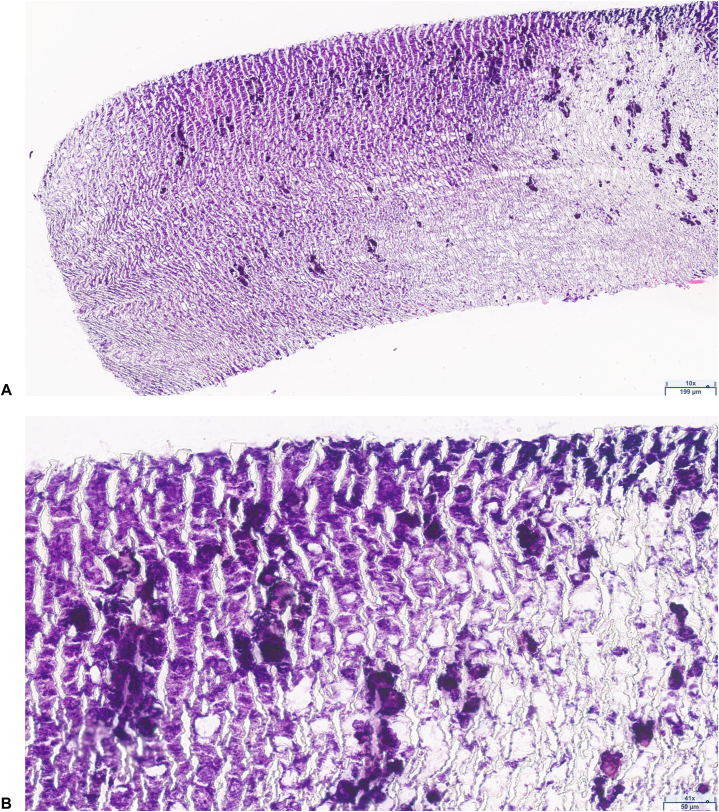
Histopathology showing plant material. **(A)** hematoxylin-eosin stain; original magnification: ×10. **(B)** hematoxylin-eosin stain; original magnification: ×41.

The biopsy resulted in foreign material consistent with plant matter (Fig 2). Upon further questioning, the patient denied any known history of trauma or inciting event, besides the cabinet corner collision, that could have led to foreign material being present in her forehead. After removal of the plant matter, the wound completely healed without further complication.

## Discussion

In many cases, patients may not be aware of retained foreign material, which can remain undetected despite examination. One study found that approximately 38% of foreign bodies were missed on initial evaluation.^2^ Additionally, the risk of retained foreign bodies from puncture wounds increases during warmer seasons, likely related to exposure of bare skin or more common outdoor recreation. People with occupations in carpentry or the garment industry are also at an increased risk.^1^ Interestingly, our patient presented for biopsy of the plant matter in a summer month, and did have previous trauma involving a cabinet.

Generally, it is advised that plant material should be removed as vegetative foreign bodies are associated with increased inflammation and risk of infection.^1^ Retained foreign bodies involving the face, neck, or deep spaces of the hands or feet should be referred for surgical removal. Of note, vegetative material is typically not visible on radiography as plant matter absorbs bodily fluids and develops a similar density as surrounding tissue.^3^ Ultrasound can detect vegetation better than radiography or computed tomography; however, the image quality is highly operator dependent.^4^

## Conflicts of interest

None disclosed.
